# Role of triggering receptor expressed on myeloid cells-1 in the mechanotransduction signaling pathways that link low shear stress with inflammation

**DOI:** 10.1038/s41598-023-31763-w

**Published:** 2023-03-21

**Authors:** Martin Liu, Anastasios Nikolaos Panagopoulos, Usama M. Oguz, Saurabhi Samant, Charu Hasini Vasa, Devendra K. Agrawal, Yiannis S. Chatzizisis

**Affiliations:** 1 Computational Cardiovascular Simulations Center, Division of Cardiovascular Medicine, Miller School of Medicine, University of Miami, 1120 NW 14th Street, Suite 1124, Miami, FL 33136 USA; 2 Cardiovascular Biology and Biomechanics Laboratory, Cardiovascular Division, Department of Internal Medicine, University of Nebraska Medical Center, Omaha, NE USA; 3 Department of Translational Research, Western University of Health Science, Pomona, CA USA

**Keywords:** Cell biology, Cardiology

## Abstract

This study sought to investigate the role of triggering receptor expressed on myeloid cells-1 (TREM-1) in the mechanotransduction signaling pathways that link low shear stress with inflammation. Human coronary artery endothelial cells, human coronary artery smooth muscle cells, and THP-1 monocytes were co-cultured and exposed to varying endothelial shear stress (ESS) conditions: low (5 ± 3 dynes/cm^2^), medium (10 ± 3 dynes/cm^2^), and high (15 ± 3 dynes/cm^2^). We showed that low ESS increased the expression of TREM-1 by the cultured cells leading to increased production of inflammatory mediators and matrix-degrading enzymes, whereas high ESS did not have a significant effect in the expression of TREM-1 and inflammatory mediators. Furthermore, TREM-1 transcriptional inhibition with siRNA in endothelial cells, smooth muscle cells, and monocytes exposed to low ESS, led to a significant reduction in the production of vascular inflammatory mediators and matrix-degrading enzymes. Additionally, we identified the transcription factors that appear to upregulate the TREM-1 gene expression in response to low ESS. To the best of our knowledge, this is the first study to investigate the pathophysiologic association and molecular pathways that link low ESS, TREM-1, and inflammation using a sophisticated in-vitro model of atherosclerosis. Future studies on animals and humans are warranted to investigate the potential of TREM-1 inhibitors as adjunctive anti-atherosclerotic therapies.

## Introduction

Low endothelial shear stress (ESS) has been proven to play a key role in inflammation and vascular biology of atherosclerosis^1^. In the presence of low shear stress, endothelial cells are activated, low density lipoprotein (LDL) is accumulated in the sub-endothelial space, monocytes transmigrate in the subendothelial space and transform to macrophages and foam cells that produce matrix-degrading enzymes and other inflammatory mediators further sustaining the atherosclerotic process. The molecular and signaling pathways that link low ESS with inflammation have not been fully elucidated.

Triggering receptor expressed on myeloid cells-1 (TREM-1) is an inflammatory mediator belonging to the immunoglobulin family and has been found to be expressed on the surface of all the cell types involved in atherosclerosis (endothelial cells, white blood cells, vascular smooth muscle cells and platelets)^2^. Studies have shown that TREM-1 plays pivotal role in the sterile inflammation of atherosclerosis^2^. One could hypothesize that TREM-1 plays an intermediate role in the association between low ESS with inflammation, however the detailed mechanotransduction signaling mechanisms that link low ESS with TREM-1 remain unknown.

The objectives of this study were the following: (i) To investigate the role of varying ESS patterns on the expression of TREM-1, pro-inflammatory molecules and matrix-degrading enzymes, (ii) To investigate the hypothesis that transcriptional inhibition of TREM-1 expression dissociates low ESS from inflammation, and (iii) To elucidate the upstream to TREM-1 mechanotransduction signaling pathways that link low ESS with TREM-1. We performed our investigations in an in-vitro multi-cellular co-culture model consisting of endothelial cells, monocytes, and smooth muscle cells, that represented the early and intermediate stages of atherosclerosis. The study design is illustrated in the Supplemental Fig. S1.

## Methods

### Description of the co-culture model

The culture model is illustrated in the Supplemental Fig. S2. The substrate of the matrix was consisted of collagen type I mixed with layers of human coronary artery smooth muscle cells (HCASMCs)^3^. The collagen matrix was coated with a single layer of Matrigel (Corning Life Sciences, Durham, NC, USA) representing the basic membrane. A single layer of human coronary artery endothelial cells (HCAECs) was plated on the Matrigel. THP-1 monocytes were added in the medium of the culture, attached to HCAECs, and allowed to migrate into the collagen matrix.

### Cells preparation

All cells were cultured according to the manufacturers’ instructions. HCAECs and HCASMCs were purchased and cultured with commercially available endothelial cell medium or smooth muscle cell medium (Cell Applications, San Diego, CA, USA). Confluent cells were sub-cultured periodically (7–8 days) using enzymatic dissociation (trypsin) at a density of 0.5–1 × 10^6^ cells per 100 mm dish. Cells of subcultures passage number #3 to #9 were used in the experiments. THP-1 monocytes (American Type of Culture Collection, Manassas, VA, USA) were cultured in RPMI-1640 supplemented with 10% fetal calf serum (10% FCS-RPMI), penicillin/streptomycin and fungizone.

### Collagen matrix

Tail tendons were extracted from rats, dissected into 1 cm-sized portions and rinsed with Tris (hydroxymethyl) aminomethane (Tris)-buffered saline (0.9% NaCl and 10 mM Tris, pH 7.5)^4^. Following dehydration and sterilization of the tendons (50%, 75%, 95%, and 100% ethanol), rat tail tendon collagen (RTTC) was extracted in a HCl solution (6 mM at 4 °C) for overnight and centrifuged (4000 rpms for 2 h). The lyophilized aliquots were weighed to measure the collagen concentration and subjected to sodium dodecylsulfate-polyacrylamide gel electrophoresis to assess the purity of the samples.

The RTTC was mixed with distilled water and Dulbecco's Modified Eagle Medium (DMEM; four times concentrated, pH 8.0) to create the collagen solution with the following characteristics: (i) Physiologic anionic strength, (ii) pH: 7.40, and (iii) Collagen concentration 0.75 mg/ml^5^. The collagen solution was used for the preparation of 0.1–0.2 mm thick collagen matrix for the experiments.

### Construction of the co-culture

For immunofluorescence staining, 100 μL of collagen solution containing HCASMCs (1 × 10^6^ cells/ml) was placed on cover slips (24 × 50 mm size). This mixture was polymerized in the incubator (37 °C, 5% CO_2_) for 30 min. Following the first incubation, Matrigel was added on top of the collagen surface (100 µl/gel) and the sample was re-incubated (37 °C, 5% CO_2_) for 1 h. Then, a single layer of HCAECs was added on top of the Matrigel-coated collagen gels (150 µl/gel, 1 × 10^6^ cells/ml) and the HCAECs were incubated (37 °C, 5% CO_2_) for 2 h to facilitate attachment. Following the endothelial attachment on the Matrigel-coated collagen gels, THP-1 monocytes were introduced on top of the endothelial cells (150 µl/gel, 2 × 10^6^ cells/ml) and allowed to migrate into the collagen matrix for 24 h.

For chromatin immunoprecipitation (ChIP) assay, real time reverse transcription PCR (RT-PCR) and enzyme-linked immunoassay (ELISA) studies, the cultures were modified as follows: 400 μl collagen-HCASMCs mixture was used, instead of 100 μl, the collagen gel was coated with Matrigel with a concentration of 400 µl/gel instead of 150 μl/gel, 450 μl of HCAECs at a concentration of 1 × 10^6^ cells/ml were used instead of 150 μl/gel, and 450 µl of THP-1 cells in a concentration of 2 × 10^6^ were used instead of 150 µl/gel.

### Exposure of co-cultures to varying shear stress patterns

The cell cultures described in 2.4 were exposed to varying shear stress patterns generated by a commercially available device (Streamer^®^; FlexCell International, Burlington, NC, USA): (i) No flow, (ii) low endothelial shear stress (low ESS: 5 ± 3 dynes/cm^2^) flow, (iii) Medium endothelial shear stress (medium ESS: 15 ± 3 dynes/cm^2^) flow, and (iv) high endothelial shear stress (high ESS: 30 ± 3 dynes/cm^2^) flow, for various time frames ranging from 30 min to 1 h.

### Small interfering RNA (siRNA) against TREM-1

To investigate the association between TREM-1, chemokines, cytokines [Interleukin (IL)-1ß, IL-6, IL-8, Monocyte chemoattractant protein-1 (MCP-1)], and matrix-degrading enzymes (MMP)-1 and -9, the cells of the co-cultures were transfected with an siRNA against TREM-1 (Santa Cruz Biotechnology, TX, USA) with the following method: HCAECs and HCASMCs were plated into culture dishes (1 × 10^6^ cells/100 mm dish at 70–80% confluency), washed with phosphate buffered saline (PBS), and cultured with 3 ml Opti-MEM medium (Invitrogen, Carlsbad, CA, USA), including lipofectamine 2000 (Invitrogen, Carlsbad, CA) and TREM-1-siRNA (200 nM; Santa Cruz Biotechnology, TX, USA) or control-siRNA for 6 h. Following the siRNA transfection, the cells were cultured with RPMI-1640 medium containing 10% FCS and penicillin/streptomycin/fungizone overnight. The transfected with TREM-1-siRNA cells were used to create the co-culture as described in 2.4. For the transfection of THP-1 monocytes, the cells were plated into culture dishes (5 × 10^6^ cells/dish) (after washing with PBS) and cultured in 3 ml of Opti-MEM medium with lipofectamine 2000 and TREM-1-siRNA (200 nM) or control-siRNA for 6 h. The transfected THP-1 cells were centrifuged and cultured in RPMI supplemented with 5% FCS and penicillin/streptomycin/fungizone overnight, and then they were introduced in the co-cultures as described in 2.4. Co-cultures transfected with scramble siRNA were used as controls of the co-cultures transfected with siRNA against TREM-1.

### Real time RT-PCR

The mRNA expression of TREM-1, chemokines (MCP-1), cytokines (IL-1ß, IL-6, IL-8) and matrix-degrading enzymes (MMP-1 and -9 and cathepsin L and S) in the cultures was performed with a real time RT-PCR system (ThermoFisher Scientific, MA, USA) with the following method: The cultured cells were exposed to various flow conditions for 1 h and then transferred into 12-well tissue plates and cultured in normal culture medium (1 ml/well) for 1 h. Collagen gels were degraded with Collagenase (0.25 mg/mL serum free RPMI; 30–60 min, 37 °C, 5% CO_2_), and the total RNA was extracted from the culture using Trizol reagent (ThermoFisher Scientific, MA, USA). Subsequently, a commercially available cDNA reverse transcription kit (High-Capacity cDNA Reverse Transcription Kit, ThermoFisher Scientific, USA) was used for the synthesis of a cDNA library using 1 µg of the extracted total RNA. The mRNA expression of TREM-1, cathepsins, cytokines, chemokines, and matrix-degrading enzymes was quantified using a commercially available real time RT-PCR system (Applied Biosystems, Waltham, MA).

### Immunoblotting

To assess the membrane-bound TREM-1 in the co-cultures, we applied immunoblotting. The co-culture models were plated on the coverslips and treated with low ESS for one hour or no flow as control. The cells were harvested after additional 5 h followed by degrading the collagen with collagenase (1 mg/mL collagenase), cell pellets were lysed with lysis buffer. Total proteins of the cell lysates were subjected to electrophoresis. Proteins were transferred to polyvinylidene difluoride (PVDF) membrane and incubated with primary anti-TREM-1 antibody (Santa Cruz, Cat # sc-293450, 1:100) or anti-ß-actin (Santa Cruz, Cat # sc-8432, 1:500) at 4 °C overnight. After washing with buffer on next day, secondary Horseradish Peroxidase-conjugated anti-mouse (Rockland Immunochemicals, Cat#: 610-1202, 1:2000) antibody was applied at room temperature for 1 h. After washing and developing with an enhanced chemiluminescent substrate (SuperSignal™ West Atto Ultimate Sensitivity Substrate), images of the immunoblotting band were obtained (Kindle Biosciences, LLC, USA).

### Immunofluorescence confocal microscopy

For the immunofluorescence confocal microscopy studies, the cultured cells (HCAECs, HCASMCs, and THP-1 cells) were plated on coverslips and exposed to various flow conditions for 1 h followed by culture in normal medium for additional 5 h. Then fixed using methanol (− 20 °C for 20 min), washed with PBS, and blocked with 5% donkey serum in PBS. Following blocking, the cells were incubated with primary rabbit anti-TREM-1 (Abcam, cat# 22586) or anti-VE-cadherin (Santa Cruz Biotechnology, cat# sc-9989) antibodies at 4 °C overnight. Next day, the cells were washed with PBS and were treated with a conjugated secondary antibody (green: anti-rabbit-Alexa 488, Invitrogen, cat# A32731; or red: anti-mouse-Alexa 568, cat# A-11004) at room temperature for 1 h in the dark. Finally, 4′,6-diamidino-2-phenylindole (DAPI) was applied to the cell nuclei for 10 min, and the cells were covered with mounting medium and visualized under confocal microscope (Zeiss LSM 800 Confocal Microscope, Germany).

### Protein quantification by ELISA

The protein levels of chemokines (MCP-1), cytokines (IL-1ß, IL-6, IL-8), cathepsins (cathepsin L and S), and MMPs (MMP-1 and -9) released by the co-cultured cells were assessed with a commercially available enzyme-linked immunoassay kit (DuoSet, ELISA Development Systems, R&D Systems, MN, USA). Following the exposure of the cultures to various flow conditions for 1 h, the cultured cells were plated into a 24-well tissue culture plates (one sample of each cover slip per well) and cultured in normal medium (500 µl/well) for 5 additional hours.

### ChIP assays for the identification of TREM-1 transcription factors

The transcription factors that binding to TREM-1 promoters were identified using the ChIP assays. The co-cultures were exposed to low ESS or no flow for 30 min each, transferred to 15 mL conical tubes and fixed (20 mL RPMI and 0.54 mL 37% formaldehyde). The specimens were washed twice (10 mL cold PBS), transferred to Eppendorf tubes with the addition of 1 mL lysis buffer [5 µL proteinase inhibitor cocktail (PIC) and 5 µL phenylmethylsulfonyl fluoride (PMSF)], incubated on ice (30 min), and placed in the freezer (− 80 °C) for 1 h. Subsequently, the specimens were homogenized (Dounce Homogenizer, Active Motif, CA, USA) and centrifuged (8000 rpm for 10 min). Following the first centrifuging, the pellets were re-suspended with 300 µL shearing buffer, supplemented with 1.75 µL PIC and 1.75 µL PMSF, sonicated 4 times (20–30 s per sonication) on ice, and were centrifuged again (13,000 rpm for 10 min). After the second centrifuging, the supernatants were collected and the DNA concentration was quantified with a spectrometer (NanoDrop 2000/2000c Spectrophotometers, ThermoFisher Scientific, USA). The collected supernatants were aliquoted and stored at − 80 °C. ChIP assay analysis was performed on the collected supernatants using a commercially available kit (ChIP-IT Express, Active Motif, Carlsbad, CA) with the following antibodies: NF-κB p65 Αb (Abcam, cat# 19870), PU.1 Ab (Santa Cruz Biotechnology, cat# sc-365208) and ATF-2 Ab (Santa Cruz Biotechnology, cat# sc-242). The specimens produced with this method were used in a polymerase chain reaction (PCR) using a commercially available kit (HotStar Taq Master Mix kit, Qiagen, USA). The primer sequences corresponding to the TREM-1 promoter (region 1 and 2) were obtained from published literature^6^ and synthesized by a commercial supplier (Life Technology, Invitrogen, CA, USA). Sequences of the primers were as following: TREM-1 promoter region 1 (− 346/+ 1), forward: TGGGCCTGACTCTCTTCACT; reverse: TGACCTAGAGGCTTCGGAAA. TREM-1 promoter region 2 (− 20/+ 261), forward: TTTCCGAAGCCTCTAGGTCA; reverse: CCCAATTCTGGGTAGAGCAG. The PCR products (7 µL/lane) were run on a 3% agarose gel and visualized under UV light.

### Statistical analysis

Statistical analyses were performed using a commercially available software (PRISM 8, GraphPad, San Diego, CA, USA). Each experiment was performed at least three times. Values were expressed as means ± standard error of mean. Real time RT-PCR data were expressed as fold change versus control. Comparison among groups were performed using a two-way ANOVA, followed by Tukey’s test to adjust for multiple comparisons between groups. *P* values of less than 0.05 were considered statistically significant.

## Results

### Effect of ESS on the expression of TREM-1, pro-inflammatory molecules and matrix-degrading enzymes

**Figure 1:**
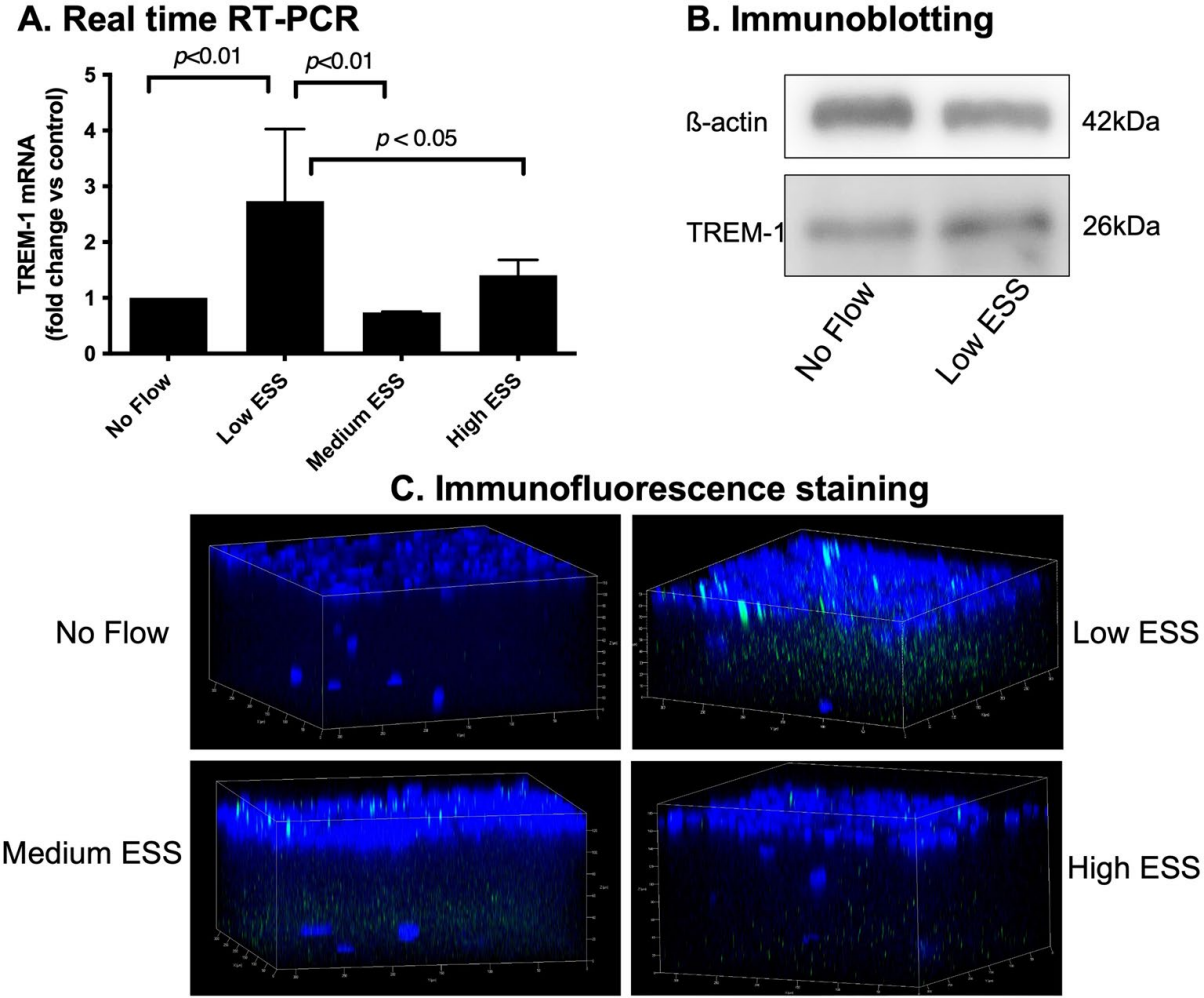
Effect of various shear stress conditions on the TREM-1 mRNA expression and protein synthesis in co-culture model. (**A**) Expression of TREM-1 mRNA in 3D co-culture cells exposed to low ESS, medium ESS, high ESS, and no flow (control). 3D co-culture of HCASMCs, HCAECs and monocytes (THP-1) cells were prepared on the cover slips and exposed to low ESS, medium ESS, high ESS, or no flow (control) for 1 h followed by additional culture for 1 h. Total RNA was extracted and expression of mRNA was quantified by real time RT-PCR as described in the “Methods”. (**B**) Immunoblotting of membrane-bound TREM-1 in 3D co-culture cells exposed to low ESS or no flow (control). 3D co-culture of HCASMCs, HCAECs and monocytes (THP-1) cells were prepared on the cover slips and exposed to low ESS or no flow (control) for 1 h followed by additional culture for 5 h. Total proteins of cell lysates were subjected to electrophoresis (10% SDS-12.5% PAGE gel) and immunoblotting to TREM-1 and ß-actin as described in the “Methods” (**B** presents the blots at 5 h. The original blots at 5 and 24 h are presented in the Supplemental Fig. S3). (**C**) Immunofluorescence staining of TREM-1 in 3D co-culture cells exposed to low ESS, medium ESS, high ESS, and no flow (control). 3D co-culture of HCASMCs, HCAECs and monocytes (THP-1) cells were prepared on the cover slips and treated with low ESS, medium ESS, high ESS, or no flow (control) for 1 h followed by additional culture for 5 h. Immunofluorescence staining to TREM-1 was then performed as described in the “Methods”. ESS: endothelial shear stress. TREM-1: triggering receptor expressed on myeloid cells-1. Data were averaged over 3 separate experiments (**A**).

**Figure 2:**
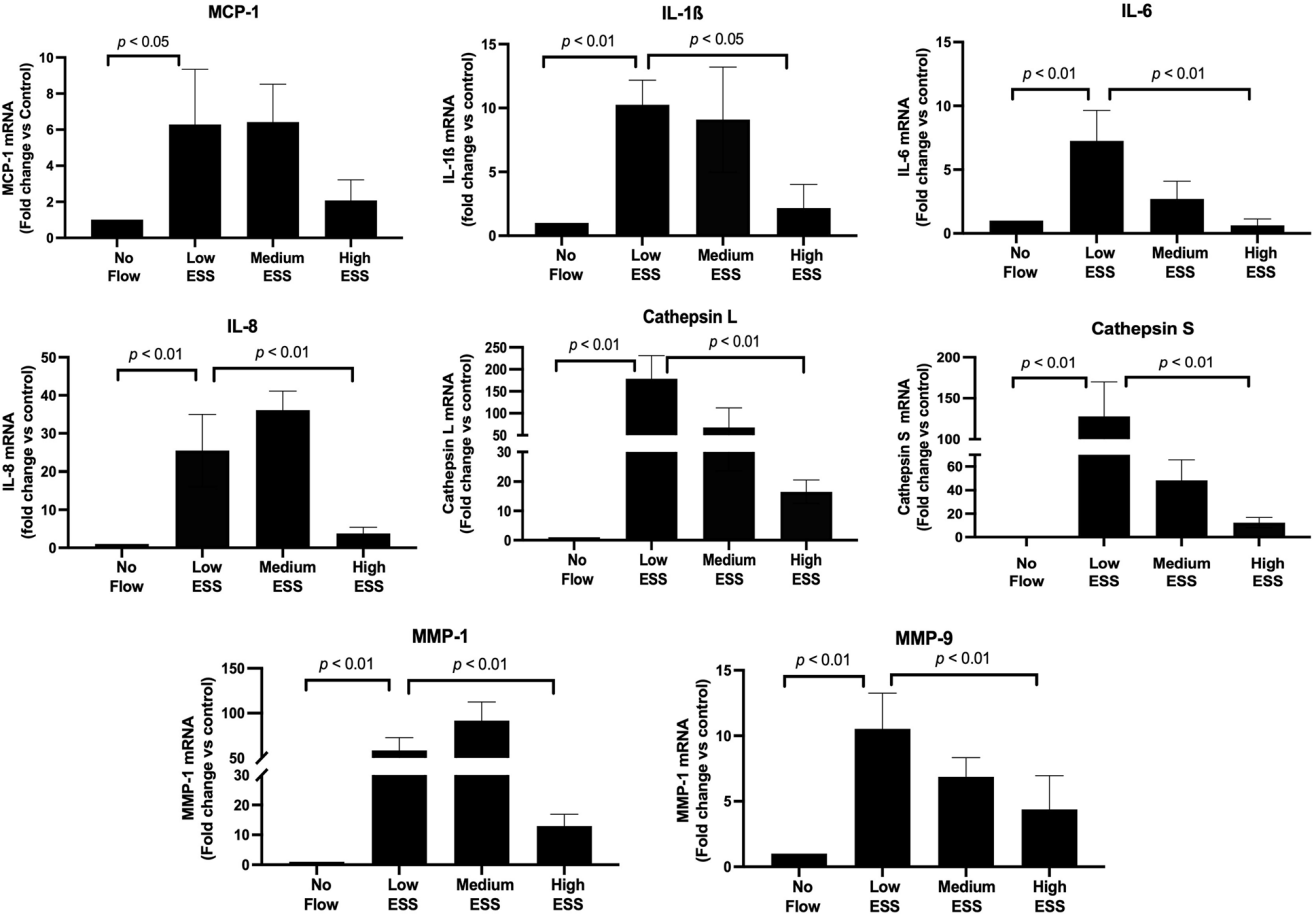
Effect of various shear stress conditions on the mRNA expression of inflammatory molecules and matrix-degrading enzymes in the co-cultures. 3D co-culture of HCAECs, HCASMCs, and monocytes (THP-1) cells were prepared on the cover slips and exposed to low ESS, medium ESS, high ESS, or no flow (control) for 1 h followed by additional culture for 1 h. Total RNA was extracted and expression of mRNA was quantified by real time RT-PCR as described in the “Methods”. IL: interleukin, ESS: endothelial shear stress, MMP: matrix metalloproteinase, MCP-1: monocyte chemoattractant protein-1, HCAECs: human coronary artery endothelial cells, HCASMCs: human coronary artery smooth muscle cells. Data were averaged over 3 separate experiments.

**Figure 3:**
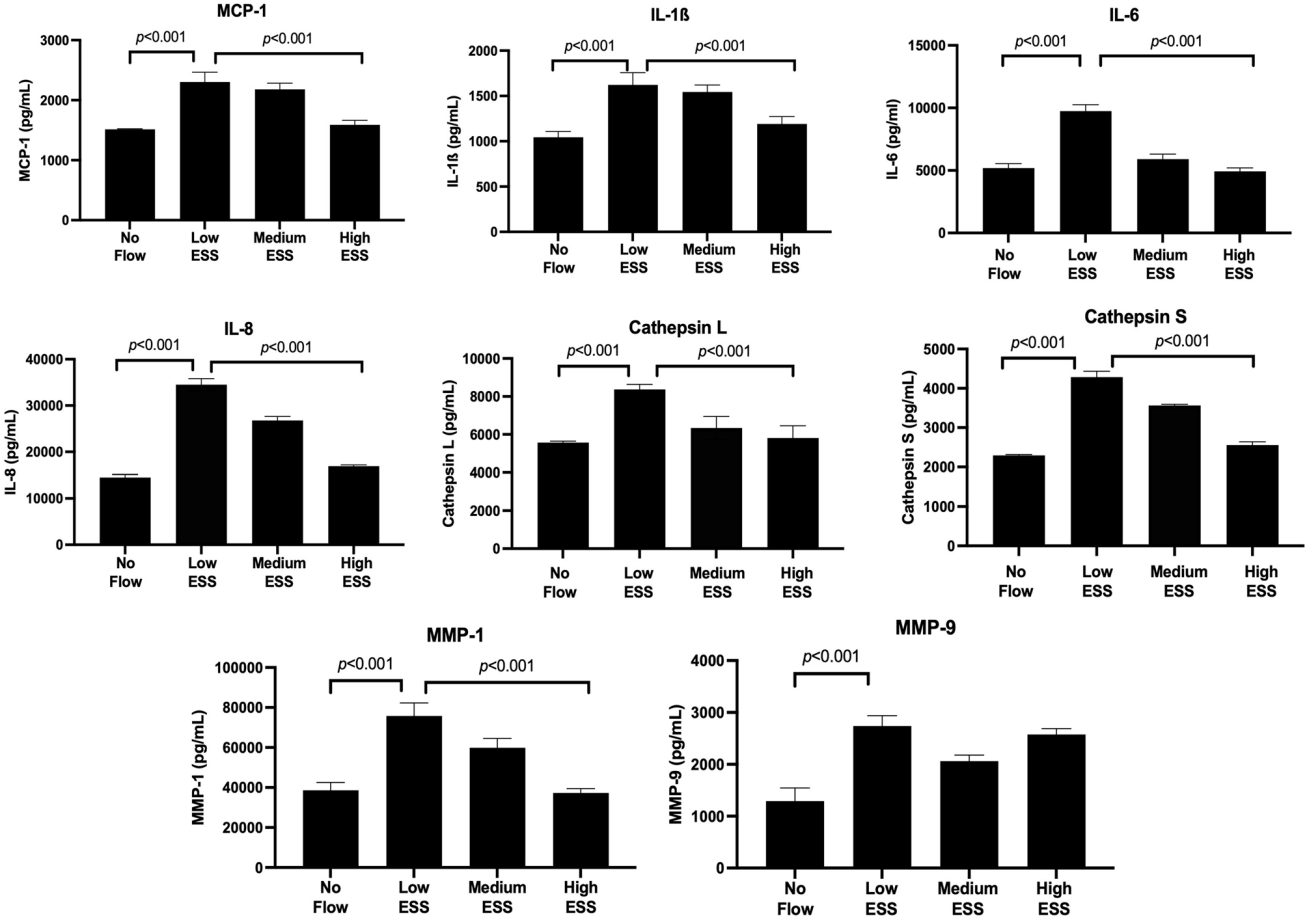
Effect of various shear stress conditions on the protein levels by ELISA of proinflammatory molecules and matrix-degrading enzymes in the medium of co-cultures. 3D co-culture of HCAECs, HCASMCs, and monocytes (THP-1) cells were prepared on the cover slips and exposed to low ESS, medium ESS, high ESS, or no flow (control) for 1 h followed by additional culture for 5 h. Amount of the proteins in the cell culture medium was quantified by ELISA as described in the “Methods”. IL: interleukin, ESS: endothelial shear stress, MMP: matrix metalloproteinase, MCP-1: monocyte chemoattractant protein-1, HCAECs: human coronary artery endothelial cells, HCASMCs: human coronary artery smooth muscle cells. Data were averaged over 3 separate experiments.

**Figure 4:**
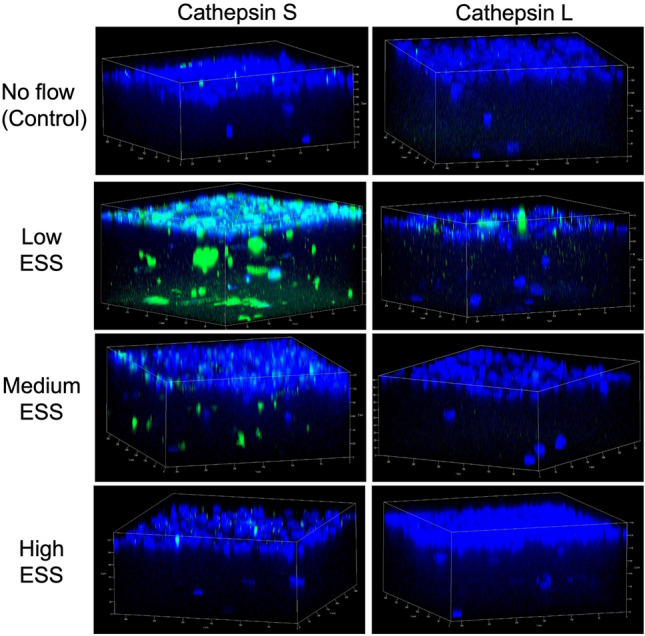
Effect of various shear stress conditions on the cathepsin protein expression in the co-cultured models. 3D co-culture of HCAECs, HCASMCs, and monocytes (THP-1) cells were prepared on the cover slips and exposed to low ESS, medium ESS, high ESS, or no flow (control) for 1 h followed by additional culture for 5 h. Immunofluorescence staining was performed as described in the “Methods”. ESS: endothelial shear stress.

Real time RT-PCR of mRNA extracted from co-cultured cells pre-exposed to varying ESS patterns showed a significant increase in the expression of TREM-1 (Fig. 1A), pro-inflammatory molecules (IL-1ß, IL-6, IL-8, MCP-1), and matrix-degrading enzymes (cathepsin L, S, as well as MMP-1, and -9) in response to low ESS, compared to no flow or high ESS (*p* < 0.01; Fig. 2). Immunoblotting and immunofluorescence confocal microscopy of the cultured cells pre-exposed to varying shear stress patterns showed that exposure of the cultures to low ESS increased the protein expression of TREM-1 by endothelial cell, monocytes, and smooth muscle cells, compared to cultured cells pre-exposed to no flow or high ESS (Fig. 1B,C, and Supplemental Fig. S3). Similar to real time RT-PCR results, ELISA assays on the medium of the co-cultures pre-exposed to varying ESS patterns showed that low ESS was associated with significantly increased production of chemokines and pro-inflammatory cytokines (MCP-1, IL-1β, IL-6, and IL-8), and matrix-degrading enzymes (cathepsins L and S, MMPs- 1 and-9; Figs. 3 and 4).

### Effect of TREM-1 inhibition with siRNA on the expression of TREM-1, Pro-inflammatory molecules, and matrix-degrading enzymes under low ESS conditions

**Figure 5:**
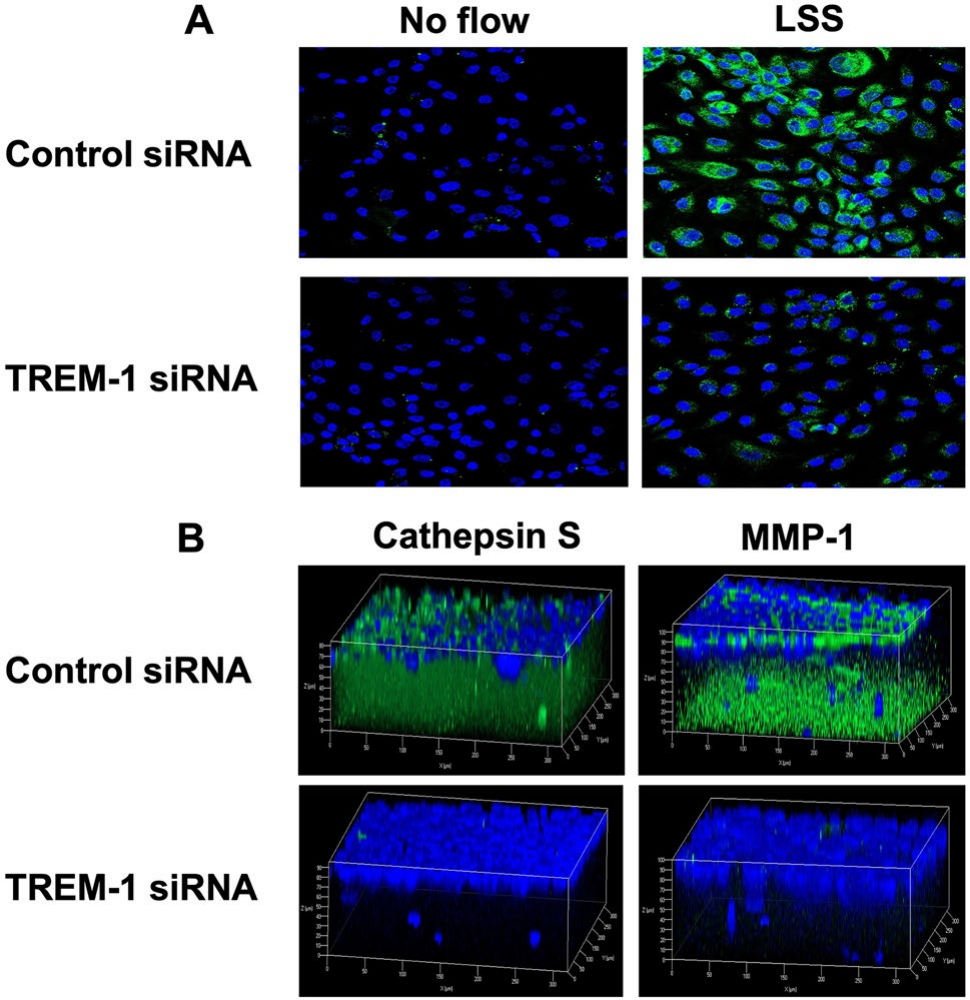
Suppression of TREM-1 protein expression by siRNA and its effect on the expression of matrix-degrading enzymes. (**A**) Suppression of TREM-1 expression by siRNA in monolayer culture of HCAECs. HCAECs were transfected with control-siRNA or TREM-1-siRNA as described in the “Methods”. Cells were then plated on coverslips and exposed to low ESS or no flow for 1 h followed by additional culture for 5 h. Cells were then fixed and immunofluorescence staining for TREM-1 was performed as described in the “Methods”. (**B**) Suppression of cathepsin S and MMP-1 protein expression in 3D co-cultures transfected with TREM-1 siRNA. HCAECs, HCASMCs and THP-1 cells were transfected with control-siRNA or TREM-1-siRNA for 6 h prior to preparing the 3D co-culture on coverslips. The cells were then exposed to low ESS for 1 h followed by additional culture for 5 h. Immunofluorescence staining for cathepsin S and MMP-1 were performed as described in the “Methods”. TREM-1: triggering receptor expressed on myeloid cells-1, siRNA: small interfering RNA, ESS: endothelial shear stress, MMP-1: matrix metalloproteinase-1, HCAECs: human coronary artery endothelial cells, HCASMCs: human coronary artery smooth muscle cells.

Immunofluorescence confocal microscopy showed that TREM-1 protein expression in monocultures of HCAECs (pre-transfected with control-siRNA) was increased in response to low ESS compared to no flow (Fig. 5A, Supplemental Fig. S4A), and this effect was attenuated with transfection of siRNA against TREM-1 into the HCAECs (Fig. 5A, supplemental Fig. S4A). In contrast, neither siRNA transfection, nor low ESS could affect VE-cadherin expression in the HCAECs (Supplemental Fig. 4B), suggesting the TREM-1-siRNA specifically targeted TREM-1 in the HCAECs without altering expressing endothelial specific biomarker (VE-cadherin).

**Figure 6:**
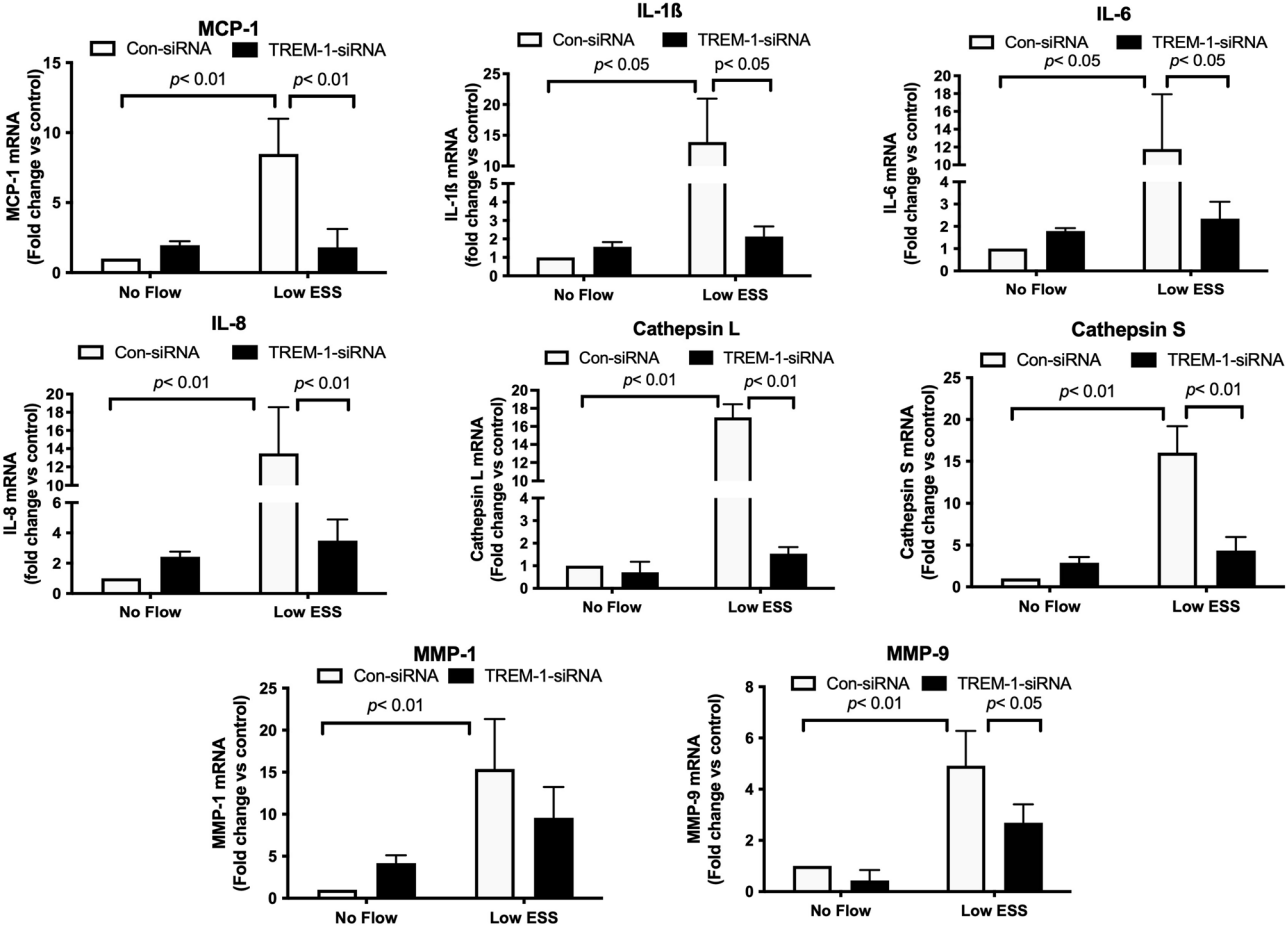
Effect of TREM-1 suppression by siRNA on the mRNA expression of pro-inflammatory molecules and matrix-degrading enzymes in 3D co-cultures exposed to low shear stress. HCAECs, HCASMCs, and THP-1 cells were transfected with control-siRNA or TREM-1-siRNA for 6 h. The 3D co-cultures were prepared on the coverslips and cultured overnight. Cells were then exposed to low ESS or no flow for 1 h followed by additional culture for 1 h. Real time RT-PCR for the quantification of mRNA was performed as described in the “Methods”. IL: interleukin, MMP: matrix metalloproteinase, MCP-1: monocyte chemoattractant protein-1, TREM-1: triggering receptor expressed on myeloid cells-1, HCAECs: human coronary artery endothelial cells, HCASMCs: human coronary artery smooth muscle cells. Data were averaged over 3 separate experiments.

**Figure 7:**
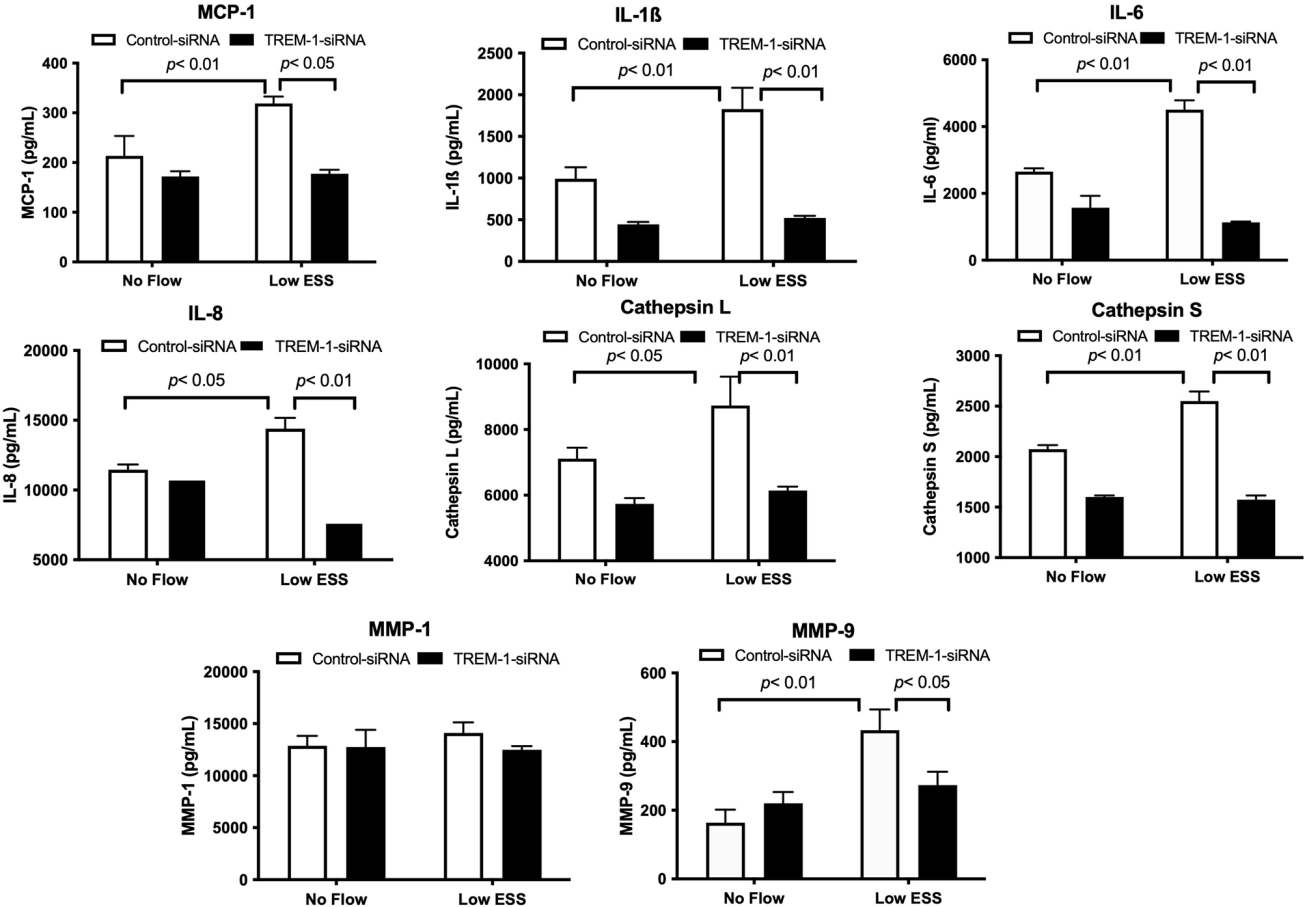
Effect of TREM-1 suppression by siRNA on the protein expression by ELISA of pro-inflammatory molecules and matrix-degrading enzymes after the exposure of the co-culture to low shear stress. HCAECs, HCASMCs, and THP-1 cells were transfected with control-siRNA or TREM-1-siRNA for 6 h. 3D co-culture was prepared on the coverslips and cultured overnight. Cells were then exposed to low ESS or no flow for 1 h followed by additional culture for 5 h. Proteins in the culture medium were quantified by an ELISA as described in the “Methods”. TREM-1: triggering receptor expressed on myeloid cells-1, siRNA: small interfering RNA, IL: interleukin, MMP: matrix metalloproteinase, MCP-1: monocyte chemoattractant protein-1, HCAECs: human coronary artery endothelial cells, HCASMCs: human coronary artery smooth muscle cells. Data were averaged over 3 separate experiments.

TREM-1-siRNA-transfected co-cultures exposed to low ESS significantly attenuated the mRNA expression of chemokines and cytokines (MCP-1, IL-1β, IL-6, IL-8), and matrix-degrading enzymes (cathepsins and MMPs) compared to control siRNA-transfected co-cultures exposed to equally low ESS conditions (Fig. 6). In accordance to real time RT-PCR results, immunofluorescence staining and ELISA on the medium of the co-cultures that were transfected with TREM-1-siRNA after exposure to low ESS showed a significant reduction in the protein level of chemokines, cytokines and matrix-degrading enzymes (Figs. 5B and 7).

### Effect of low ESS on the activation of TREM-1 transcription factors

**Figure 8:**
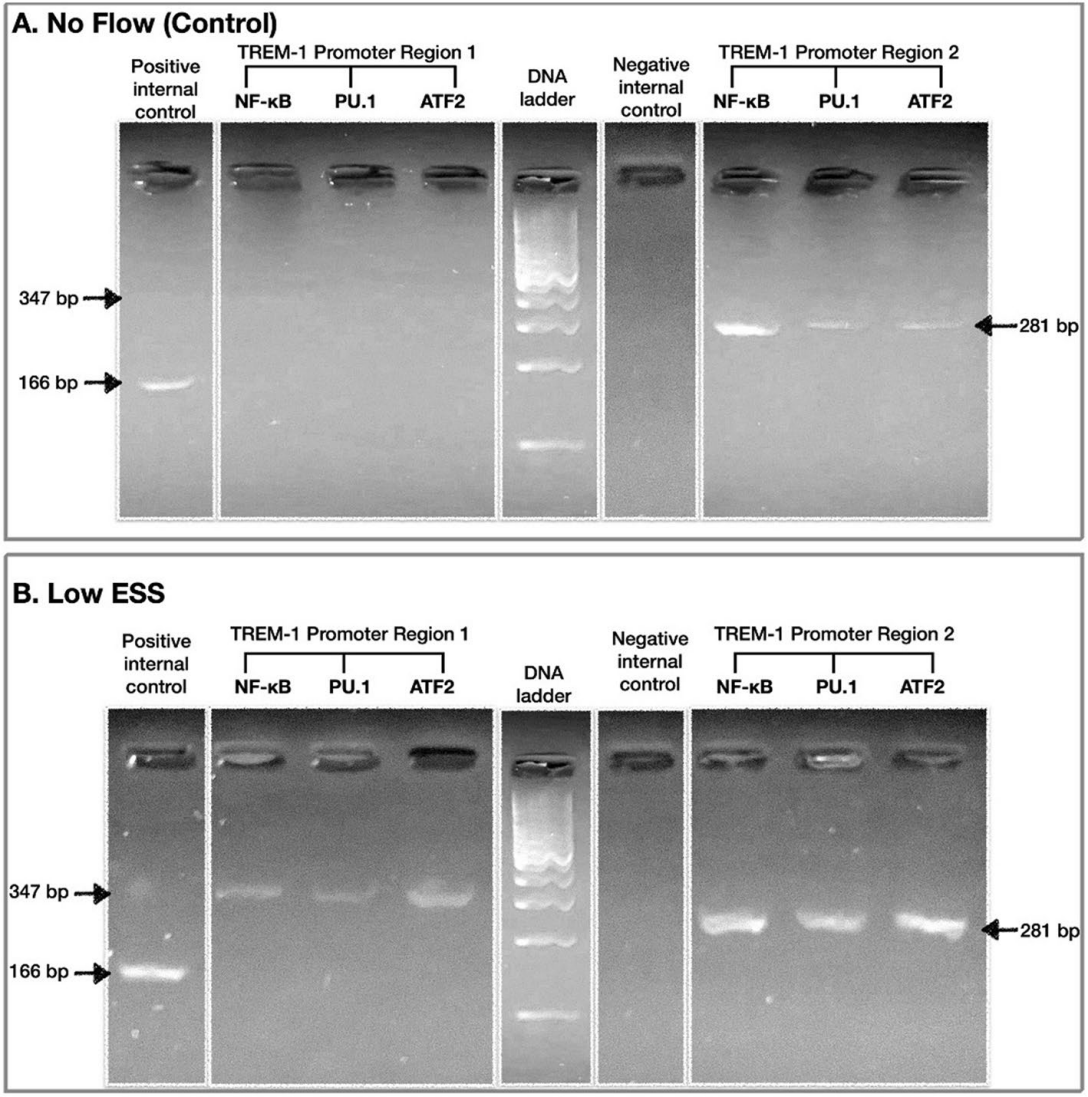
Comparison of the binding of transcription factors (NF-κB, PU.1 and ATF2) to the TREM-1 promoter regions 1 and 2 between low ESS and no flow conditions. 3D co-cultures of HCAECs, HCASMCs, and monocytes (THP-1) were prepared on the coverslips and cultured overnight. Cells were then exposed to low ESS or no flow for or 30 min. ChIP assay was then performed as described in the “Methods”. ESS: endothelial shear stress, TREM-1: triggering receptor expressed on myeloid cells-1, NF-κΒ: Nuclear factor kappa B, ATF2: Activating transcription factor 2.

ChIP assay investigation using the co-cultured cells exposed to different flow conditions (low shear stress and no flow conditions) showed enhanced binding of the transcription factors NF-κB, PU.1 and ATF2 to the TREM-1 promoter region 2 under low ESS conditions, compared to no flow (Fig. 8). Additionally, low ESS conditions induced the binding of the abovementioned transcription factors to TREM-1 promoter region 1, compared to no flow. Of note, under low ESS conditions, the binding of transcription factors to TREM-1 promoter region 2 was greater compared to promoter region 1.

## Discussion

In the present study, we used a co-culture model consisting of endothelial cells, monocytes and smooth muscle cells, to investigate the intermediate role of TREM-1 in the molecular pathways that link low shear stress with inflammation. To delineate the role of TREM-1 between low ESS and inflammation, we eliminated the confounding role of LDL, since LDL has been found to promote inflammation through the TREM-1 pathways independently of shear stress^2^. We showed that low ESS increased the expression of TREM-1 by the cultured cells leading to increased production of inflammatory mediators and matrix-degrading enzymes, whereas high ESS did not have a significant effect in the expression of TREM-1 and inflammatory markers. Furthermore, TREM-1 transcriptional inhibition with siRNA in endothelial cells, smooth muscle cells, and monocytes exposed to low ESS, led to a significant reduction in the production of vascular inflammatory mediators and matrix-degrading enzymes. Additionally, we identified NF-κB, PU.1 and ATF2 as the transcription factors that may upregulate the TREM-1 gene expression in response to low ESS. To the best of our knowledge, this is the first study to investigate the pathophysiologic association and molecular pathways that link low ESS, TREM-1 and inflammation using a sophisticated in-vitro model of atherosclerosis.

### Association of low ESS, TREM-1, and inflammation

Low ESS is a well-known inducer of vascular inflammation. Low ESS is mechanotransduced via surface mechanoreceptors on endothelial cells and through various intracellular pathways leading to the activation of transcription factors and increased expression of several pro-inflammatory genes^7–9^. The activation of pro-inflammatory genes leads to endothelial cell damage and migration of inflammatory cells into the intima, which augments the synthesis and activation of MMPs and cathepsins in the atherosclerotic plaque^7–10^. These enzymes contribute to extracellular matrix degradation in the arterial wall and fibrous cap, leading to further transmigration and accumulation of inflammatory cells, such as monocytes and smooth muscle cells in the atherosclerotic plaque, ultimately promoting atherosclerotic plaque progression and destabilization.

TREM-1 is an activating receptor expressed on the surface of endothelial cells, immune cells (neutrophils, monocytes macrophages) and vascular smooth muscle cells^2^. TREM-1 activation amplifies the vascular inflammatory response through increased production of pro-inflammatory molecules, production of matrix-degrading enzymes and recruitment of inflammatory cells. Several studies have highlighted the implication of TREM-1 activation in various pathophysiologic processes that contribute to atherosclerosis and its complications such as endothelial dysfunction, inflammatory lipid accumulation, plaque destabilization and increased thrombogenicity^2^. However, to date, no study has focused on the association of low ESS with TREM-1 activation. In this study, we demonstrated that the mechanotransduction of low ESS in endothelial cells leads to activation of TREM-1 transcription factors, which in turn upregulate the expression of the TREM-1 gene leading to increased production of pro-inflammatory molecules and matrix-degrading enzymes. Previous studies have showed that the transcription factors NF-κB, PU.1 and ATF2 are associated with the modulation of the TREM-1 gene transcription^6,11^, however, to date, no study has investigated the association of these transcription factors with TREM-1 gene transcription in conditions of low ESS in an atherosclerosis-relevant model. In the present study, we build upon the state-of-the-art by showing that NF-κB, PU.1 and ATF2 are the transcription factors that mediate the TREM-1 gene transcription in condition of low ESS.

### TREM-1 inhibition with siRNA dissociates low ESS from inflammation

Previous studies have reported that suppression of TREM-1 by RNA interference significantly impaired the inflammatory response to oxidized LDL in macrophages^12^, and that genetic deletion (trem-1 knockout mice) or pharmacological inhibition (LR12 peptide) of TREM-1 significantly reduced the development of atherosclerosis throughout the vascular tree and lessened plaque inflammation in mice^13^. In this study, we used an siRNA against TREM-1 to suppress the expression of TREM-1 in cultured cells exposed to low ESS. Consistent with previous reports^2,12,13^, our results showed that transfection of the co-cultures with siRNA decreased the expression of TREM-1 by the co-cultured cells, and this decrease was associated with reduced expression of cytokines and matrix-degrading enzymes, despite the ongoing low ESS stimulus. This observation supports the hypothesis that TREM-1 inhibition dissociates low ESS from inflammation.

### Future studies

We showed that low ESS activates TREM-1 transcription factors in endothelial cells, however, the specific mechanism of low ESS mechanotransduction in endothelial cells is unclear. One could hypothesize that TREM-1 could directly mechanotransduce low ESS in endothelial cells, however this remains to be proven in future investigations. Additionally, the potential ligand that mediates TREM-1 activation in conditions of low ESS remains to be determined. HMGB-1, a known TREM-1 ligand, that is up-regulated by endothelial cells in condition of low ESS and could potentially serve as the local TREM-1 activator^2^. Also, the signaling mechanisms downstream to the activation of mechanoreceptors and upstream to the activation of TREM-1 transcription factors (NF-κB, PU.1 and ATF2), as well as the cell types expressing the TREM-1 and transcription factors are unclear and warrant further investigation. Another aspect that warrants further investigation is that potential synergistic effect of LDL on TREM-1 expression and inflammation as well as on the upregulation of mechanoreceptors**.** One would expect LDL to have a synergistic effect with low ESS on the expression of TREM-1, pro-inflammatory mediators, and mechanoreceptors. To investigate further the additional effects of LDL in the co-culture, the cultured models should be supplemented with LDL particles and cultured under conditions of low ESS. Finally, the effects of low ESS in the activation of TREM-1 in cells other than endothelial cells (monocytes and smooth muscle cells) warrants further investigation.

### Limitations

Our study had several limitations: First, the collagen matrix used in the co-cultures lacked fibronectin and proteoglycans, which are present in normal human vascular wall. Second, the density of HCASMCs in the co-cultures might not resemble the *in-vivo* conditions in the human vascular wall. Third, the potential TREM-1 ligand mediating TREM-1 activation in the cultured cells was not identified. Fourth, even though adding LDL to the cultures would make them more relevant to human atherosclerosis, we elected not to add LDL in order to eliminate the confounding effect of LDL on TREM-1 and inflammation. Fifth, we did not identify the specific intracellular molecular pathways downstream to the activation of mechanoreceptors by low ESS.

## Conclusion

Our investigations in an easy-to-make, low-maintenance and reproducible model, which is representative of the advanced stages of atherosclerosis in humans, showed that low ESS increased the expression of inflammatory molecules and matrix-degrading enzymes through the activation of TREM-1. The transcription factors associated with the low ESS-induced TREM-1 activation are NF-κB, PU.1 and ATF2. Notably, TREM-1 mRNA inhibition with siRNA could dissociate low ESS from vascular inflammation and plaque destabilization. Future studies on animals and humans are warranted to investigate the potential of TREM-1 inhibitors as adjunctive anti-atherosclerotic therapies.

## Supplementary Information

Supplementary Figures.

## Data Availability

Correspondence and requests for materials should be addressed to Y.S.C.
