# Supplementary material for: Role of triggering receptor expressed on myeloid cells-1 in the mechanotransduction signaling pathways that link low shear stress with inflammation
Source: Sci Rep. 2023 Mar 21;13:4656. doi: 10.1038/s41598-023-31763-w (PMC10030555; doi:10.1038/s41598-023-31763-w)

Role of Triggering Receptor Expressed on Myeloid Cells-1 in the Mechanotransduction Signaling Pathways that Link Low Shear Stress with Inflammation

Martin Liu, MD, PhD[1,2*]; Anastasios Nikolaos Panagopoulos, MD[2*]; Usama M. Oguz, MD[1,2*]; Saurabhi Samant, MBBS[2]; Charu Hasini Vasa, MBBS[1,2]; Devendra K. Agrawal, PhD[3]; Yiannis S. Chatzizisis, MD, PhD[1,2]

[1]Computational Cardiovascular Simulation Center, Division of Cardiovascular Medicine, Miller School of Medicine, University of Miami, Miami, FL
[2]Cardiovascular Biology and Biomechanics Laboratory, Cardiovascular Division, Department of Internal Medicine, University of Nebraska Medical Center, Omaha, NE
[3]Department of Translational Research, Western University of Health Science, Pomona, CA

* The first three authors contributed equally

Supplemental Figures

Supplemental Figure S1. Study design.
TREM-1: triggering receptor expressed on myeloid cells-1, siRNA: small interfering RNA, HCAECs: Human coronary artery endothelial cells, HCASMCs: Human coronary artery smooth muscle cells

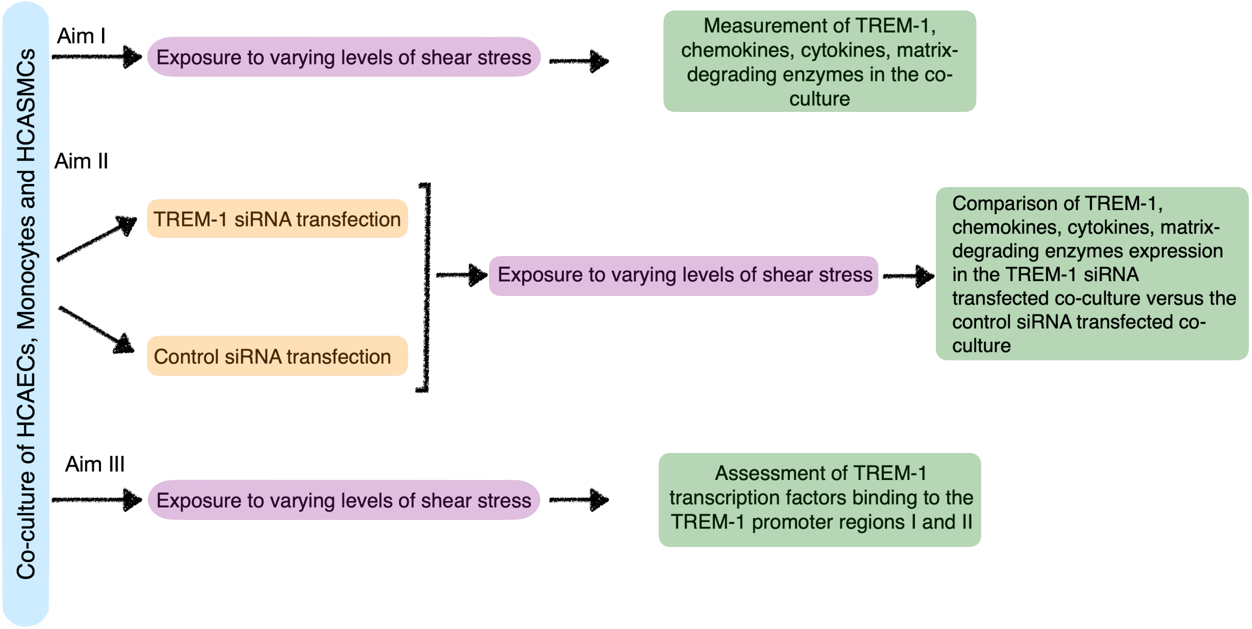

Supplemental Figure S2. Demographic illustration of the co-culture of HCAECs, monocytes and HCASMCs.

HCAECs: Human coronary artery endothelial cells, HCASMCs: Human coronary artery smooth muscle cells.

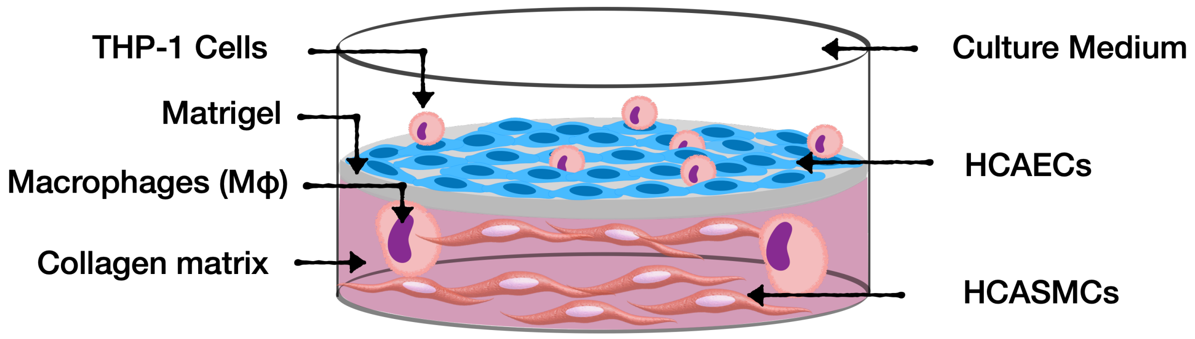

Supplemental Figure S3. Original immunoblotting images of membrane-bound TREM-1 and ß-actin in 3D co-culture cells exposed to low ESS or no flow (control). 3D co-culture of HCASMCs, HCAECs and monocytes (THP-1) cells were prepared on the cover slips and exposed to low ESS or no flow (control) for 1 hour followed by additional culture for 5 and 24 hours, respectively. Total proteins of cell lysates were subjected to electrophoresis (10% SDS-12.5% PAGE gel) and immunoblotting to TREM-1 and ß-actin as described in the methods. As shown in the blots, in addition to the expected TREM-1 at molecular size of 26-30 kDa, fusion proteins of TREM-1 were also detected at approximately 40-50 kDa as expected according to the anti-TREM-1 antibody information from the manufacturer. TREM-1: triggering receptor expressed on myeloid cells-1, HCASMCs: human coronary artery smooth muscle cells, HCAECs: human coronary artery endothelial cells, ESS: endothelial shear stress.

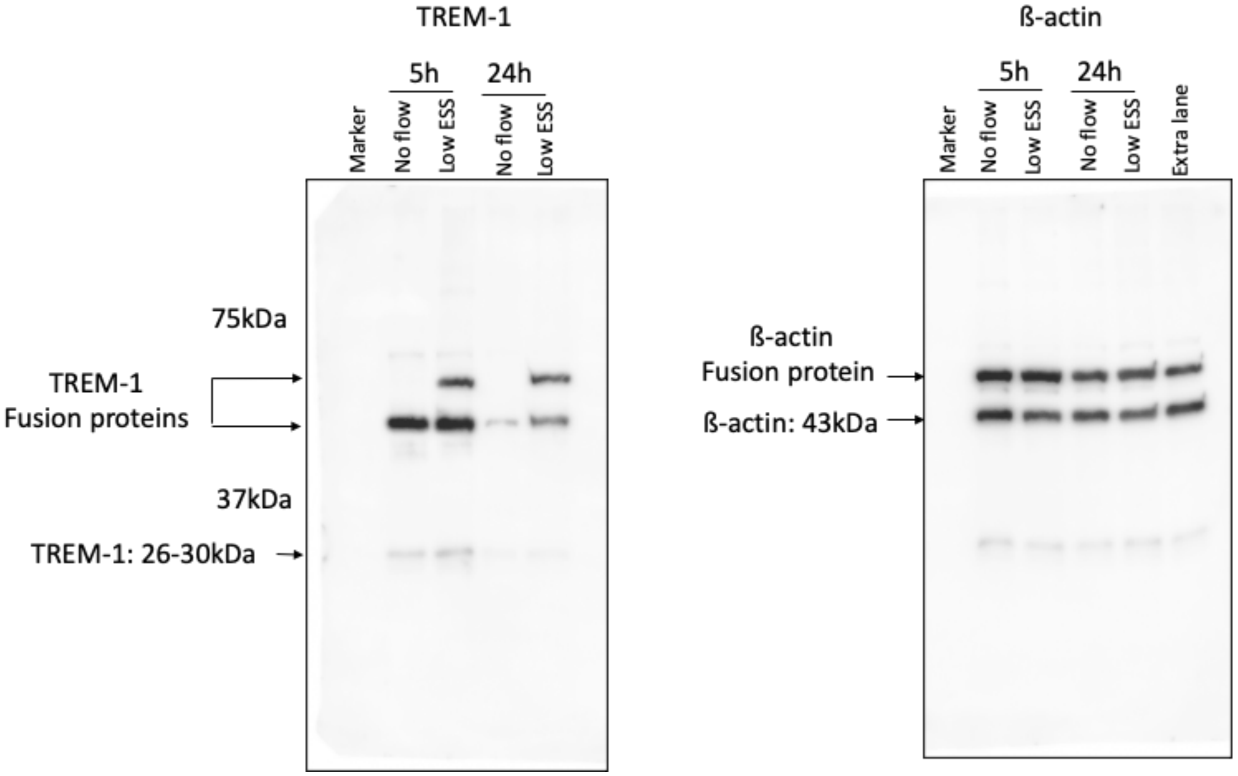

Supplemental Figure S4. Suppression of TREM-1 expression by siRNA in the monolayer culture of endothelial cells. A. Effect of shear stress condition on the expression of TREM-1 in HCAECs transfected with control-siRNA or TREM-1-siRNA. B. Effect of low ESS or high ESS on the expression of VE-cadherin in HCAECs transfected with control-siRNA or TREM-1-siRNA. HCAECs were transfected with control-siRNA or TREM-1-siRNA for 6 hours. Cells were then trypsinzed and plated on the coverslips and cultured overnight. Cells were then exposed to low ESS, high ESS, or no flow for one hour followed by additional culture for 5 hours in the medium. Cells were then fixed and immunostained for TREM-1 and VE-cadherin as described in the methods. Note that low ESS but not high ESS dramatically stimulated expression of TREM-1 in the cells transfected with control-siRNA, and it was significantly suppressed in the cells transfected with TREM-1-siRNA. In contrast, VE-cadherin expression was not affected. TREM-1: triggering receptor expressed on myeloid cells-1, siRNA: small interfering RNA, ESS: endothelial shear stress. Data presented was one representative of 3 separate experiments.

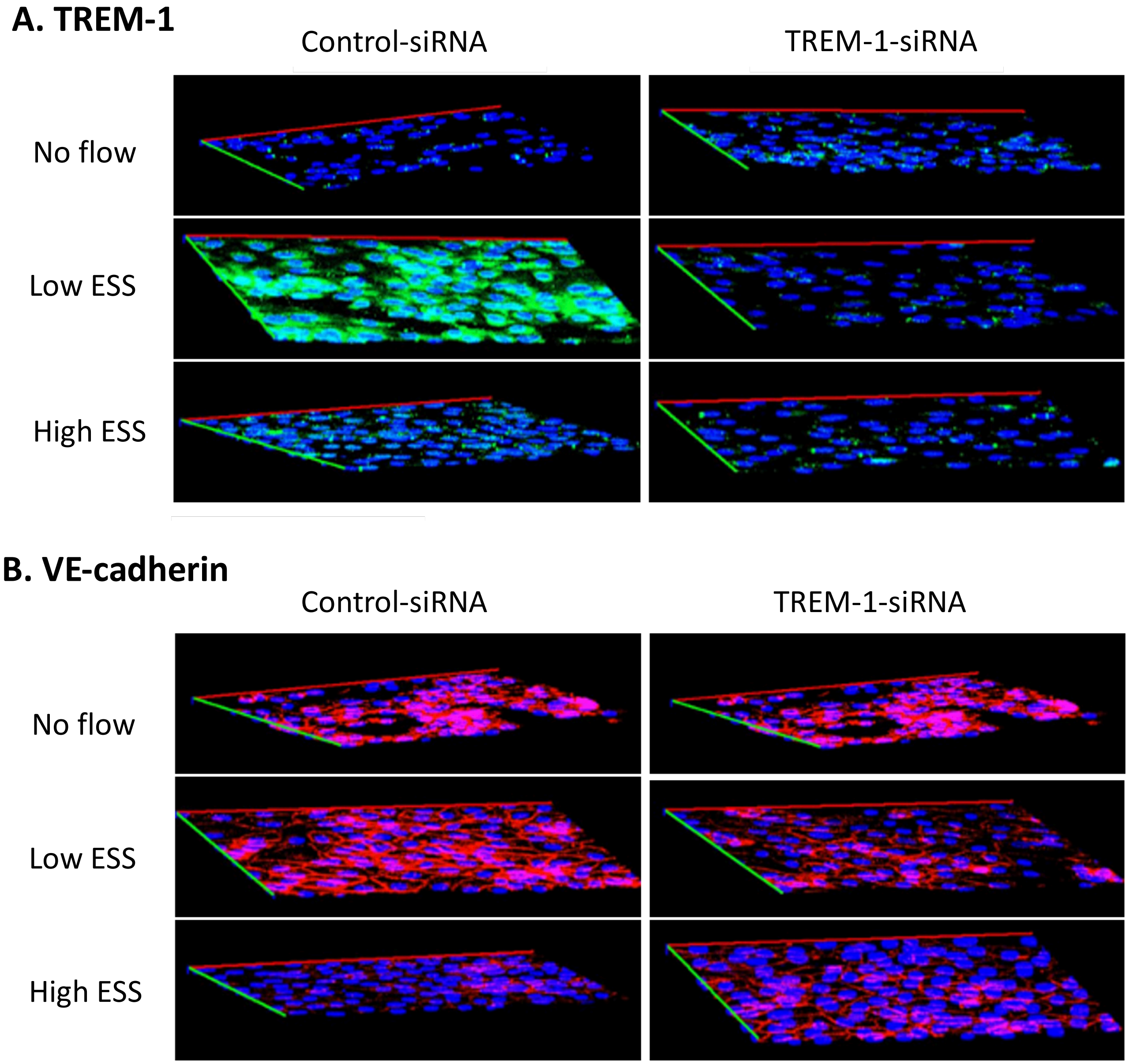

Supplement: Supplementary file 1 — Supplementary Figures. [file 41598_2023_31763_MOESM1_ESM.pdf]
